# A Novel, Reliable and Highly Versatile Method to Evaluate Different Prion Decontamination Procedures

**DOI:** 10.3389/fbioe.2020.589182

**Published:** 2020-10-29

**Authors:** Hasier Eraña, Miguel Ángel Pérez-Castro, Sandra García-Martínez, Jorge M. Charco, Rafael López-Moreno, Carlos M. Díaz-Dominguez, Tomás Barrio, Ezequiel González-Miranda, Joaquín Castilla

**Affiliations:** ^1^Center for Cooperative Research in Biosciences (CIC bioGUNE), Basque Research and Technology Alliance (BRTA), Bizkaia Technology Park, Derio, Spain; ^2^Atlas Molecular Pharma S. L., Bizkaia Technology Park, Derio, Spain; ^3^IKERBASQUE, Basque Foundation for Science, Bilbao, Spain

**Keywords:** transmissible spongiform encephalopathy, prion, decontamination, PMSA, *in vitro* propagation

## Abstract

Transmissible spongiform encephalopathies (TSEs) are a group of invariably fatal neurodegenerative disorders. The causal agent is an aberrantly folded isoform (PrP^Sc^ or prion) of the endogenous prion protein (PrP^C^) which is neurotoxic and amyloidogenic and induces misfolding of its physiological counterpart. The intrinsic physical characteristics of these infectious proteinaceous pathogens makes them highly resistant to the vast majority of physicochemical decontamination procedures used typically for standard disinfection. This means prions are highly persistent in contaminated tissues, the environment (surfaces) and, of great concern, on medical and surgical instruments. Traditionally, decontamination procedures for prions are tested on natural isolates coming from the brain of infected individuals with an associated high heterogeneity resulting in highly variable results. Using our novel ability to produce highly infectious recombinant prions *in vitro* we adapted the system to enable recovery of infectious prions from contaminated materials. This method is easy to perform and, importantly, results in highly reproducible propagation *in vitro*. It exploits the adherence of infectious prion protein to beads of different materials allowing accurate and repeatable assessment of the efficacy of disinfectants of differing physicochemical natures to eliminate infectious prions. This method is technically easy, requires only a small shaker and a standard biochemical technique and could be performed in any laboratory.

## Introduction

Transmissible spongiform encephalopathies (TSEs) or prion diseases are a group of invariably fatal neurodegenerative disorders that affect humans and a wide variety of mammals (Aguzzi and Calella, 2009). The main event in prion pathogenesis is the conformational conversion of the cellular prion protein (PrP^C^) into a pathological conformer (PrP^Sc^) (Prusiner, 1982a). These two isoforms differ only in their three-dimensional structures and also functionally in that the pathological isoform is both neurotoxic and able to induce conformation change of the normal cellular counterpart, PrP^C^, to the pathological isoform, PrP^Sc^ (Edgeworth et al., 2010). The latter, upon misfolding, contains a higher content of β-sheets and this makes it prone to aggregation, insolubility and partial resistance to treatment with proteases (Kocisko et al., 1994; Saborio et al., 2001). For many decades the resistance of pathogenic prions to standard decontamination procedures attracted the attention of part of the scientific community due to the public health implications.

Transmissibility of scrapie was first demonstrated in 1936 (Cuille and Chelle, 1936) and subsequent studies focused on identifying the causative agent, which was believed initially to be a virus. However, this led to the discovery of the prion protein and the extraordinary resistance of pathogenic prions to inactivation. The first recognition of this was prior to the knowledge that prions were responsible for TSEs when part of a Scottish sheep flock suffered from scrapie after being given a vaccine against louping ill virus prepared from sheep brain homogenates treated previously with 0.35% formalin (Gordon, 1946; Giles et al., 2017). Further studies demonstrated that the scrapie agent in sheep brain was active after treatment with up to 40% formalin (Stamp et al., 1959; Pattison, 1965) and could withstand extreme temperatures (Stamp et al., 1959; Pattison and Millson, 1961) and UV irradiation (Alper et al., 1967) also, suggesting it might be devoid of nucleic acid. Nuclease digestion repeatedly failed to inactivate the infectious agent (Prusiner et al., 1978; Prusiner et al., 1980b) as well as most other procedures targeting nucleic acids. Therefore, treatments that modify amino acid residues were then tested to assess if they could inactivate prions. Initially, proteinase K and trypsin (Hunter and Millson, 1967; Prusiner et al., 1981b) were shown to reduce the titer of the scrapie agent in a concentration-dependent manner. Some chemical modifications also inactivated the scrapie agent, including treatment with diethyl pyrocarbonate (Prusiner et al., 1980a), butanedione and phenylmethylsulfonyl fluoride (PMSF) (Prusiner, 1982b). Although weak chaotropic agents failed to inactivate scrapie, strong chaotropic agents such as thiocyanate, guanidinium, and trichloroacetate are effective (Prusiner et al., 1981a). Similarly, exposure to high concentration of denaturants, like urea, decreased prion infectivity (Hunter, 1969) as did treatment with SDS or phenol (Prusiner et al., 1980a), all of which supported the “protein only” hypothesis.

The resistance of prions to conventional methods of pathogen decontamination (Taylor, 2000) leads to persistence in the environment and increased risk of transmission including to other mammalian species. While many prion diseases are apparently not transmissible to humans, bovine spongiform encephalopathy (BSE) provides a perfect example of prion zoonosis and highlights the hazard posed by prions to humans (Pattison, 1998). This novel form of human prion disease, known as variant Creutzfeldt-Jakob disease (vCJD), which was directly related to BSE-contaminated meat consumption, became the first zoonotic human prion disease (Pattison, 1998; Scott et al., 1999) and give rise to an unprecedented health and food crisis that highlighted the risk of deficient decontamination of prions. However, vCJD is not the only acquired prion disease in humans. Iatrogenic CJD (iCJD) refers to the transmission of prions via inadvertent medical exposure, emphasizing that efficient prion decontamination processes are necessary to avoid transmission to health care providers and patients (Gibbs et al., 1969; Duffy et al., 1974; Simpson et al., 1996; el Hachimi et al., 1997; Brown and Farrell, 2015; Bonda et al., 2016).

Despite traditional protocols being ineffective against prion infectivity (Taylor, 2000), the World Health Organization (WHO) and the Centre for Disease Control and Prevention (CDC) of the United States of America published guidelines for prion decontamination for commonly used instruments (WHO, 1999; CDC, 2019). The advice was to incinerate all instruments after single use but as this was not feasible the WHO suggested procedures for both heat-resistant and heat-sensitive instruments. However, efficient protocols are not practical for routine decontamination and proved to be damaging to several surgical devices. On the contrary, those that are harmless for the instrumentation barely eliminate prions (Sehulster, 2004; Brown et al., 2005). Therefore, novel decontamination methods capable of abolishing prion infectivity and allowing the reuse of expensive, heat-sensitive medical devices remain elusive (Crotti, 2020).

To assess the efficacy of prion decontamination/inactivation procedures accurate, reliable evaluation methods are required and these have, traditionally, used prion-infected brain homogenates of different species (Tateishi et al., 1988; Peretz et al., 2006). Although some of these procedures were evaluated using cell culture (Supattapone et al., 2001), bioassays were an unavoidable step to evaluate decontamination in terms of prion infectivity. Prions retain their infectivity *in vivo* when bound to metal surfaces (Zobeley et al., 1999; Flechsig et al., 2001) so research has focused primarily on novel decontamination procedures to ensure the safety of stainless steel medical instruments (Fichet et al., 2004). Contaminated steel wires have been widely used to model the contamination of surgical material and have achieved more representative results than whole brain homogenates, cell cultures (Edgeworth et al., 2011) or *in vitro* methods such as Real-time quaking-induced conversion (RT-QuIC) (Hughson et al., 2016). As *in vitro* prion propagation methods become more reliable, with respect to recombinant prion infectivity, bioassay is considered increasingly unnecessary thereby paving the way for prion decontamination assessment methodologies based exclusively on prion-contaminated steel wires and *in vitro* protocols (Belondrade et al., 2016; Mori et al., 2016). Methods using infected brain homogenates can be inaccurate as, in most cases, wet homogenates are employed despite clear evidence that dehydration can increase the persistence of infectious prions in the environment (Yuan et al., 2018). Although assays based on steel wires addressed this issue, these methods can only evaluate the effect of different decontaminants or procedures on prions attached to steel surfaces and do not reproduce the effects of different surface materials on the infectivity of adhered prions nor the efficacy of any decontamination procedure.

In the present study, we exploit PMSA (Protein Misfolding Shaking Amplification), a novel technique for propagation of infectious recombinant prion *in vitro* (Erana et al., 2019), and the ability of recombinant prions to bind to surfaces comprised of different materials to establish an affordable method to evaluate prion-specific decontaminants. Contrary to conventional decontaminant assessment methodologies using brain homogenates, PMSA reduces variability by using highly reproducible recombinant infectious prions. Beads of different materials, representing some of the common surfaces in clinical or laboratory settings, were investigated for their ability to adsorb prions, and PMSA was adapted to propagate the adsorbed prions. This allowed us to measure the efficacy of disinfectants with different distinct physicochemical properties to abolish prion propagation capacity from a diverse range of materials’ surfaces as proof of concept for this novel assay without the requirement for expensive specialist equipment.

## Materials and Methods

### Generation of Prion-Coated Beads

We used a prion strain, obtained in previous experiments [termed *L-seeded-PMSA (1)* in (Erana et al., 2019) and abbreviated as Sst01], derived from the mammalian strain CWD-Vole109I and propagated *in vitro* by Protein Misfolding Cyclic Amplification (PMCA) using a substrate of recombinant bank vole I109 PrP (rec-VoPrP) complemented with dextran sulfate (Fernández-Borges et al., 2017a). It was adapted to the PMSA methodology by serial passaging ensuring it retained its strain properties which included biochemical features and *in vivo* and *in vitro* propagation (Erana et al., 2019). Four different types of beads; glass (BioSpec Products Inc.), zirconia/silica (BioSpec Products Inc.), stainless steel (Luis Aparicio S.L.), all (1 mm), and PTFE (Teflon^®^, 2 mm) (Luis Aparicio S.L.), were coated with the chosen recombinant prion. Prior to coating, the beads were rinsed by vortexing three times with phosphate buffered saline (PBS), each lasting at least 4 h with intense shaking (700 rpm), to eliminate impurities or dust from their surfaces that could influence the efficiency of prion adsorption. After a final rinse with sterile water the beads were dried completely in a stove (42°C) overnight. For the coating, approximately 1 ml of the *Sst01* inoculum was added to each 2 ml tube containing approximately 500 mm^2^ of beads. The tubes were placed on a mixer and incubated for 1–2 h with gentle agitation (40 rpm) at room temperature (20–25°C). Subsequently, the supernatant was removed and the beads subjected to 24 h PMSA using 800 μl of fresh rec-VoPrP substrate in 2 ml tubes as described below. After PMSA, the supernatant was removed and the beads rinsed by vortexing twice with PBS and twice with sterile water and dried completely in a stove (42°C) overnight prior to being stored at room temperature. Each batch of prion-coated beads was always tested before their use to ensure proper coating and reproducibility of the results in terms of propagation capacity of prions adhered to beads. For that, at least four beads of the recently coated batch were used as seeds in four different 24 h PMSA reactions using 800 μl of fresh rec-VoPrP substrate in 2 ml tubes as described below. The samples were afterward digested with proteinase K, submitted to electrophoresis and total protein staining in order to monitor the presence of rec-PrP^res^. Only those batches of prion-coated beads able to give rise to rec-PrP^res^ in all the replicates were considered valid for the decontamination assays, to ensure reproducibility of the methodology.

### Decontamination of Prion-Coated Beads

Beads coated with infectious prions were subjected to different procedures commonly used for decontamination of equipment, materials and surfaces (Table 1). To assess the effectiveness of temperature-based decontamination protocols, the coated beads were subjected to autoclaving for 20 min either at 121°C, the traditional temperature but insufficient for complete prion inactivation, or at 134°C, effective for prion decontamination according to WHO recommendations (WHO, 1999). Similarly, to assess the effect of ultraviolet irradiation coated beads were exposed to direct UV light at 254 nm for 1 h with orbital shaking to achieve complete exposure. For the evaluation of chemical disinfection procedures, the beads were submerged in the following solutions for 1 h and subjected to gentle agitation at room temperature: commercial bleach (37% chlorine) (Henkel); sodium hydroxide (NaOH) 1N (Fisher Scientific); Virkon^TM^ at 1% (Zotal); and a mixture of sodium dodecyl sulfate (SDS) 1% (Sigma-Aldrich) and acetic acid (AcO) 0.5% (Thermo Fisher Scientific). In addition, a set of beads was treated with PBS as a negative control. In all cases, after the decontamination procedure the beads were rinsed by vortexing twice with PBS and twice with distilled water.

**TABLE 1:** Physicochemical decontamination treatments selected to evaluate the novel assay developed for the assessment of potential prion inactivation procedures.

**Type**	**Decontaminant**	**Conditions**	**References**
Physical	Autoclave at 121°C	Exposition of the beads to 121°C for 20 min.	Known to be insufficient for prion inactivation (WHO, 1999).
	Autoclave at 134°C	Exposition of the beads to 134°C for 20 min.	Recommended for decontamination of prions (WHO, 1999).
	UV	Exposition of the beads to a UV (254 nm) lamp for 1 h.	Known to be insufficient for prion inactivation (Bellinger-Kawahara et al., 1987).
Chemical	Bleach	Incubation of the beads in commercial bleach (approximately 37 g hypochlorite per L) for 1 h with gentle shaking.	Recommended for decontamination of prions (WHO, 1999).
	NaOH	Incubation of the beads in 1 N NaOH solution for 1 h with gentle shaking.	Recommended for decontamination of prions (WHO, 1999).
	Virkon^TM^	Incubation of the beads in 1% Virkon^TM^ solution for 1 h with gentle shaking.	Commonly used virucidal agent. Effect on prions unknown.
	AcO-SDS	Incubation of the beads in a solution with 2% SDS detergent together 1% AcOH at room temperature for 1 h with gentle shaking.	Partially effective against prions (Peretz et al., 2006).

To investigate whether addition of detergents could improve the efficiency of prion inactivation, prion-coated Teflon^®^ beads were treated for 1 h with bleach or NaOH solutions, both containing 1% Triton-X-100 (Sigma-Aldrich) and subjected to mild shaking. Under the same PMSA conditions (see below), using 1 and 2 beads with reaction times of 1, 2, and 4 h and their respective positive and negative controls, the presence or absence of rec-PrP^res^ propagation was evaluated by proteinase K digestion, electrophoresis and total protein staining.

### Preparation and Purification of Recombinant PrP for the PMSA Substrate

The bacterial expression of bank vole I109 recombinant prion protein (rec-VoPrP) was achieved using a pOPIN E expression vector prepared using standard molecular biology procedures, as described previously (Elezgarai et al., 2017; Fernández-Borges et al., 2017a; Fernández-Borges et al., 2017b). Briefly, the oligonucleotides 5′ AGGAGATATACCATGAAGAAGCGGCCAAAGCCTGG 3′ and 5′ GTGATGGTGATGTTTGGAACTTCTCCCTTCGTAGTA 3′ (Sigma-Aldrich) were used to amplify the target sequence, i.e., the ORF of the *PRNP* gene from bank vole I109, from genomic DNA. The pOPIN E vector was digested with *Nco*I and *Pme*I (Fermentas) restriction enzymes, and the insert was introduced into the vector by homologous recombination using the In-Fusion cloning method. The final construct was transformed in *E. coli* Rosetta (DE3) competent bacteria using standard molecular biology procedures, and the expression of the protein of interest was induced upon addition of isopropyl β-D-1-thiogalactopyranoside (IPTG) at 1 mM (Gold BioTechnology) to the LB broth (Pronadisa) used for culture. The bacterial pellet obtained from each liter of culture, after centrifugation was resuspended in 50 ml of lysis buffer containing 50 mM Tris-HCl (Fisher Scientific), 5 mM EDTA (Sigma-Aldrich), 1% Triton X-100 (Sigma-Aldrich), 1 mM PMSF (Sigma-Aldrich) and 100 μg/ml lysozyme (Sigma-Aldrich), and then incubated with 100 U/ml DNase (Sigma-Aldrich) and 20 mM MgCl_2_ (Sigma-Aldrich) for 30 min with stirring at 200 rpm at room temperature. The resultant lysate was centrifuged at 8,500 *g* and 4°C for 1 h, and the resulting pellet, containing bacterial inclusion bodies, was resuspended in 50 ml of washing buffer [20 mM Tris-HCl, 150 mM NaCl (Sigma-Aldrich), 1 mM EDTA, 1% Sarkosyl (Sigma-Aldrich)]. After an additional centrifugation at 8,500 *g* at 4°C for 1 h, the new pellet was dissolved in 6 ml of inclusion buffer [20 mM Tris-HCl, 0.5 M NaCl and 6 M GdnHCl (Sigma-Aldrich)] and incubated overnight at 37°C with stirring to break down inclusion bodies and solubilize the recombinant protein. A final centrifugation at 8,500 *g*, at 4°C for 1 h and filtration thought a 0.20 μm-pore membrane (Corning) were performed prior to purification. At this point, the first fraction of 10 μl was collected to monitor the process, which is called the Lysis fraction. Although the recombinant protein did not contain a His-tag, the purification step was performed using a histidine affinity column coupled to Aktä FPLC equipment (GE Healthcare), taking advantage of the natural His residues present in the octapeptide repeat region of PrP. The affinity column was first equilibrated with 10 column volumes (CV) of Binding buffer [20 mM Tris-HCl, 500 mM NaCl, 5 mM imidazole (Sigma-Aldrich) and 2 M GdnHCl], then the lysate obtained from the bacterial pellet was loaded into the column, collecting the second 10 μl fraction, the Loading fraction, from the flow through. Once loaded, the column was washed with 15 CV of Binding buffer, from which the next 10 μl fraction was collected, the Washing fraction. Finally, the protein was eluted in 30 ml of elution buffer containing 20 mM Tris-HCl, 500 mM NaCl, 500 mM imidazole and 2 M GdnHCl, from which the final fraction, named Elution was taken, and then completely denatured by addition of GdnHCl to a final concentration of 6 M. The four fractions collected during the purification process were precipitated by adding an equivalent to the 85% of the volume of sample of ice-cold methanol, incubation for 30 min at −20°C and centrifugation at maximum speed for 20 min at room temperature. After drying all the methanol from the precipitated samples, these were resuspended in loading buffer (NuPage 4X, Invitrogen) diluted 1:3 (v:v) in PBS and submitted to electrophoresis and total protein staining (Supplementary Figure 1). The final concentration of the protein was adjusted to 25 mg/ml, using the absorbance at 280 nm measured with a NanoDrop^TM^ (Thermo Fisher Scientific), by concentrating the eluted solution using 10 kDa centrifugal filter units (Millipore), aliquoted and stored at −80°C until required. The stability of the protein has been evaluated in terms of its capacity to sustain prion propagation efficiency using serially diluted seeds. According to this internal tests, the protein stored at −80 or −20°C, without further thawing and freezing cycles maintains the *in vitro* propagation capacity for at least 1 year, although may keep sufficient quality for propagation likely indefinitely. At lower storage temperatures, such as or 4°C, the propagation capacity decays with time, but we have determined that it is suitable for PMSA at least for 1 month.

### Preparation of PMSA Substrate

For the substrate, the prepared rec-VoPrP protein was thawed, diluted 1:5 in PBS (Fisher Scientific) and dialyzed against PBS (1 L for 100 μl of protein) for 1 h. The dialyzed protein was then diluted 1:10 in conversion buffer (CB) containing 0.15 M NaCl and 1% Triton X-100 and complemented with 0.5% dextran sulfate 6,500–10,000 kDa (Sigma-Aldrich). In contrast to the purified protein, which is conserved at 6 M of GdnHCl, substrates are devoid of denaturants and show shorter half-life. For this study they were used immediately after the first thawing, keeping the conveniently aliquoted substrate at −80°C for about a month. In such storage conditions, substrates have been found to have maximum propagation efficiency up to 1 year after preparation at least. The same substrates conserved at 4°C, without freezing have been found to be stable and show the same propagation efficiency for 4 days maximum.

### Protein Misfolding Shaking Amplification (PMSA)

The PMSA protocol used was based on that published previously (Erana et al., 2019) with slight modifications to optimize it for bead-adsorbed prions. Briefly, prion-coated beads of different materials (1 or 5 for glass beads, zirconia/silica beads and stainless steel beads, and 1 or 2 for PTFE beads) were placed in clean, labeled 2 ml tubes (Fisher Scientific) after the decontamination procedures (see above). Fresh substrate (500 μl) was added to each tube and they were subjected to a single PMSA round at 39°C and 700 rpm shaking. The duration of the *in vitro* propagation was determined for each set of conditions (type of surface and number of beads) using prion-coated beads not subjected to any decontamination procedure to allow discrimination between weak and strong decontamination procedures. Moreover, it was used to prove that the efficiency of the propagation was linearly dependent upon the number of beads and the duration of the PMSA. Thus, three durations (**t1**; time at which propagation is still undetectable, **t2**; time at which propagation is detectable but has not reached plateau and **t3**; time at which prion propagation has reached plateau) were selected for each setting: 1 h, 2 h, and ≥ 4 h for reactions containing a single bead of any material or 2 PTFE beads (due to their larger size). Additional timings included: 30 min, 1 h, and ≥ 2 h for reactions containing 5 Zirconia/silica or stainless steel beads, and 15 min, 30 min, and ≥ 2 h for reactions containing five glass beads. Each reaction was performed in triplicate and was accompanied by two positive controls (i.e., non-decontaminated prion-loaded beads at the shortest and the longest times used) and one negative control (i.e., non-loaded beads at the longest time used) to check for cross-contamination or spontaneous misfolding. The use of distinct amounts of beads and different reaction times was selected because it could help to establish a scale for the different decontamination treatments, revealing weak disinfectants as opposed to strong ones. Presumably, the weak disinfectants would be able to reduce propagation with the lowest amount of beads and at the shortest reaction times, while the stronger ones should be able to inhibit prion propagation in all conditions. To evaluate the effect of the different decontamination treatments on prion propagation, just intermediate (t2) and longest (t3) PMSA reaction times were taken into consideration, since t1, a time point in which propagation should not be detected, was included as a control for the process to check that the propagation kinetics were the expected ones for each type of bead. In all cases, positive controls (prion-coated beads not subjected to any decontamination procedure that were washed in the same way than the treated ones) for t1 and t3 were included and negative controls (uncoated beads of the same type) were also included for t3 to evaluate cross-contamination or spontaneous misfolding.

### Proteinase K Digestion and Total Protein Staining

The PMSA-derived products were digested with proteinase K (Roche) and subjected to electrophoresis and staining of total protein to assess the presence of proteinase K-resistant misfolded PrP (rec-PrP^res^) in each sample. Briefly, 400 μl of each PMSA product were transferred to 1.5 ml Eppendorf tubes and proteinase K added to a final concentration of 25 μg/ml. The tubes were incubated at 42°C for 1 h and then centrifuged at 19,000 *g* at 4°C for 15 min. The supernatant was discarded carefully without disturbing the pellet and the pellet was washed with 500 μl of PBS and centrifuged again at 19,000 *g* for 5 min at 4°C. After removing all the PBS, the pellet was resuspended in 15 μl of loading buffer NuPage 4X (Invitrogen) diluted in PBS; at this point samples could be stored at −20°C until required. For electrophoresis, digested samples, together with non-digested controls, were boiled at 100°C for 10 min and loaded in 4–12% acrylamide gels (Invitrogen) for 1 h 20 min at 70 V for 10 min at 110 V for the next 10 min and 150 V for 1 h. The gel was then transferred to a glass bucket and total protein stained using BlueSafe (NzyTech) for at least 1 h at room temperature with gentle rocking.

### Statistical Analysis of Prion Decontamination Studies

A grouped analysis using a two-way ANOVA with Bonferroni’s post-test was performed to assess the effect of each decontaminant in different materials based on detection or absence of proteinase K-resistant misfolded recombinant PrP, comparing with absence of decontaminant (control situation). GraphPad Prism 5^®^ software was used for statistical analyses. No measurement was excluded for statistical analysis. For all analyses, *p* ≤ 0.05 was considered significant. Data displayed in Tables 2, 3 represent mean ± SD, excluding those cases for which the SD was 0. Statistically significant differences are also indicated in Tables 2, 3 with the following symbols: ^×^*p* < 0.05, ^××^*p* < 0.01, and ^∼^*p* < 0.001.

**TABLE 2:** Summary of the results obtained for rec-PrP^res^ propagation in PMSA from the different prion-coated beads treated with the chosen different decontamination procedures.

	**Autoclave 121°C**		**Autoclave 134°C**		**UV**		**NaOH**		**Bleach**		**Virkon^TM^**		**AcO-SDS**	
**Material**	**t2**	**t3**	**t2**	**t3**	**t2**	**t3**	**t2**	**t3**	**t2**	**t3**	**t2**	**t3**	**t2**	**t3**
Glass	6/6	6/6	0/6^∼^	4/6 (±0.52)	6/6	6/6	4/6 (±0.52)	6/6	0/6^∼^	2/6^∼^ (±0.52)	3/6^×^ (±0.55)	6/6	2/6^∼^ (±0.52)	6/6
Zr-Si	6/6	6/6	5/6 (±0.41)	6/6	5/6 (±0.41)	6/6	3/6^×^ (±0.55)	6/6	0/6^∼^	0/6^∼^	5/6 (±0.41)	6/6	5/6 (±0.41)	6/6
Steel	0/6^∼^	0/6^∼^	0/6^∼^	0/6^∼^	5/6 (±0.41)	6/6	0/6^∼^	0/6^∼^	0/6^∼^	0/6^∼^	2/6 (±0.52)	6/6	4/6 (±0.52)	6/6
Teflon^®^	3/6 (±0.55)	6/6	0/6^∼^ (±0.55)	2/6^∼^ (±0.52)	3/6 (±0.55)	6/6	3/6 (±0.55)	6/6	1/6^×^ (±0.41)	5/6 (±0.41)	0/6^∼^	0/6^∼^	3/6 (±0.55)	6/6

*Number of replicate tubes subjected to PMSA showing rec-PrP^res^ by electrophoresis and total protein staining/total number of replicates. Presence of rec-PrP^res^ indicates incomplete inactivation of the rec-PrP^res^ adsorbed to the surface of the beads. t2 refers to the shortest PMSA reaction time in which rec-PrP^res^ was detected in the absence of any decontamination treatment but propagation has not reach a plateau, and t3 refers to the shortest PMSA reaction time in which rec-PrP^res^ signal was considered to have reached plateau which is considered to be the maximum propagation limit. The numbers in parenthesis show the mean ± SD, excluding those cases for which SD was 0. Statistically significant differences with respect to untreated controls are also indicated with the following symbols: ^×^*p* < 0.05, ^××^*p* < 0.01, and ^∼^*p* < 0.001.*

**TABLE 3:** Summary of the results obtained for rec-PrP^res^ propagation in PMSA from the prion-coated Teflon^®^ beads treated with NaOH and bleach with Triton X-100 added.

	**NaOH**		**NaOH + Triton X-100**		**Bleach**		**Bleach + Triton X-100**	
**Material**	**t2**	**t3**	**t2**	**t3**	**t2**	**t3**	**t2**	**t3**
Teflon^®^	3/6 (± 0.55)	6/6	0/6^∼^	0/6^∼^	1/6^×^ (±0.41)	5/6 (±0.41)	0/6^∼^	0/6^∼^

*Number of replicate tubes subjected to PMSA showing rec-PrP^res^ by electrophoresis and total protein staining/total number of replicate tubes is represented for each treatment. Presence of rec-PrP^res^ indicates incomplete inactivation of the seeds adsorbed to the surface of the beads. t2 refers to the shortest PMSA reaction time in which rec-PrP^res^ can be detected in the absence of a decontamination treatment but propagation has not reach a plateau, and t3 refers to the shortest PMSA reaction time in which the rec-PrP^res^ signal reached plateau, which is considered to be the maximum propagation limit. The numbers in parenthesis show the mean ± SD, excluding those cases for which SD was 0. Statistically significant differences with respect to untreated controls are also indicated with the following symbols: ^×^p < 0.05, ^××^*p* < 0.01, and ^∼^p < 0.001.*

## Results

### Development of a New Method for Recombinant Prion Propagation *in vitro* Based on Coated Beads Used as Seed

**FIGURE 1:**
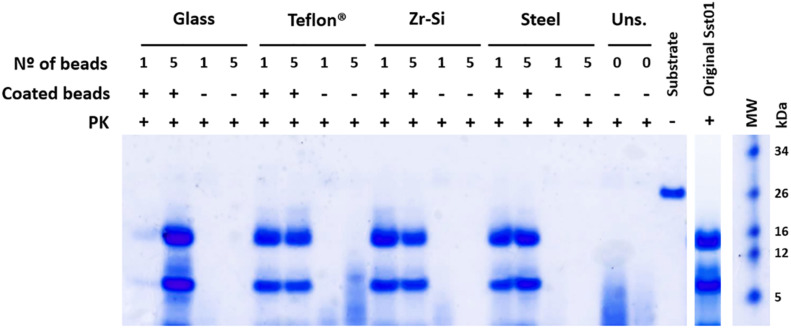
Efficient recombinant prion propagation by PMSA using different prion-coated beads as seed. Electrophoresis and total protein staining of the PMSA products obtained, using either 1 or 5 recombinant prion-coated beads of different materials as seeds, showing rec-PrP^res^ after treatment with Proteinase K (PK). Prion-coated beads of glass, zirconia/silica, stainless steel and Teflon^®^ were used as seeds and the same beads without prion coating were also submitted to the same process as controls of cross-contamination or spontaneous rec-PrP^res^ formation. Unseeded (Uns.) reactions without beads and without any seeding were also used for this purpose. All prion-coated beads were able to give rise to rec-PrP^res^, showing the same electrophoretic patterns as the original Sst01 strain with predominant ∼16 and ∼9 kDa fragments. No cross-contamination or spontaneous formation of rec-PrP^res^ was detected in any of the unseeded reactions either with or without beads. A sample of the substrate rec-PrP without PK digestion is also shown as reference for the size of the undigested rec-PrP. MW, Molecular weight marker.

All prion-coated beads made of different materials were able to give rise to Proteinase K-resistant misfolded recombinant PrP (rec-PrP^res^) upon PMSA and showed the same electrophoretic mobility pattern and PK-resistant fragments (∼16, 9, and 2 kDa) as the original Sst01 (Figure 1). The same beads not coated with rec- PrP^res^ were unable to generate these products after PMSA as denoted by electrophoresis. In some cases small molecular weight fragments were present and most likely represented spontaneous formation of amorphous PrP aggregates from the substrate. This can also be observed in unseeded reactions in the absence of beads.

### Characterization of the Propagation Capacity of Each Type of Coated Bead by PMSA

**FIGURE 2:**
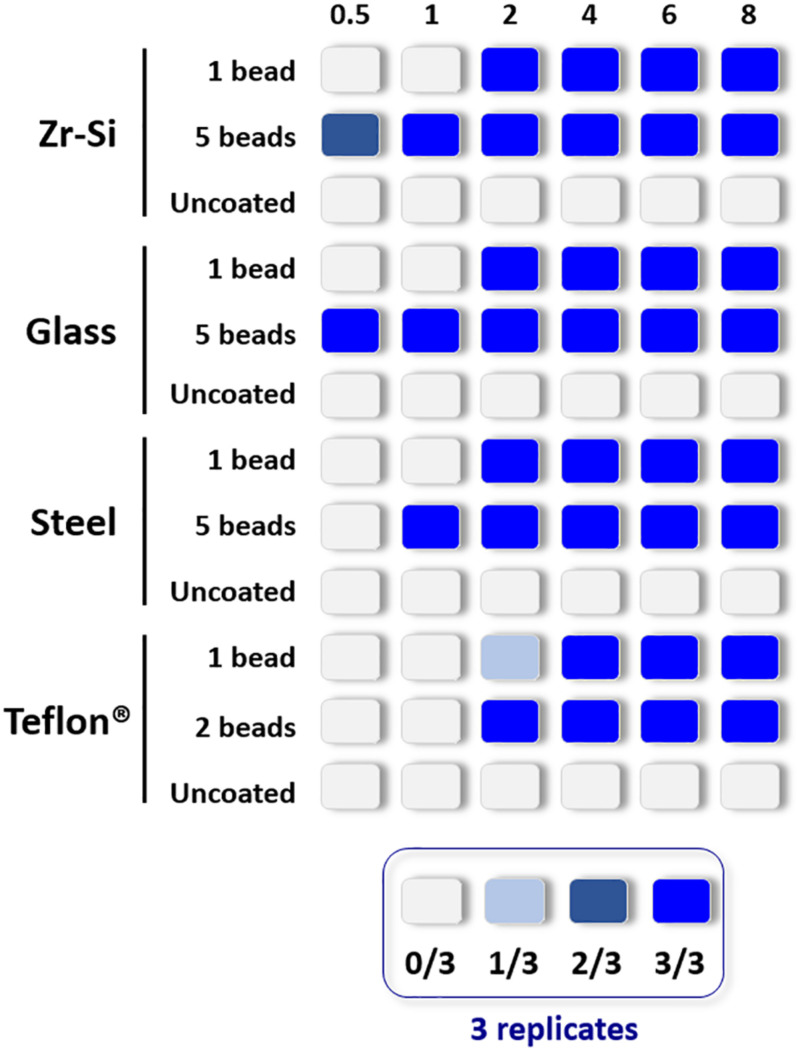
Representation of the propagation capacity of each type of prion-coated bead at different PMSA reaction times. In order to characterize the propagation kinetics of each prion-coated bead type and determine the best propagation time window for evaluation of decontamination treatments, either 1 or 5 prion-coated beads were used as seeds (except for Teflon^®^ beads for which 1 or 2 beads were used) and triplicate samples submitted to PMSA from 30 min to 8 h. The maximum number of uncoated beads of each type were also used in the same conditions as controls for cross contamination or spontaneous rec-PrP^res^ generation. The figure represents with different shades of blue the number of replicate tubes in which rec-PrP^res^ was detected for each PMSA time as indicated in the legend. Rec-PrP^res^ was detected for zirconia-silica (Zr-Si) and glass beads as early as 30 min when using 5 beads while the prion-coated steel beads required 1 h and the 2 prion-coated Teflon^®^ beads 2 h. When a single bead of each type was used 3 out of 3 replicates showed detectable rec-PrP^res^ at 2 h except for the Teflon^®^ bead that required 4 h to reach the same result.

The propagation capacity of each type of prion-coated bead was examined at different PMSA reaction times and results are summarized in Figure 2 (raw data available at Supplementary Figure 2). The most readily amplified rec-PrP was that adsorbed to zirconia-silica beads, for which rec-PrP^res^ was detected at 30 min reaction time with either 1 or 5 coated beads. Prion-coated glass beads gave the next most rapid result, 1 bead gave rise to rec-PrP^res^ at 2 h and 5 beads as early as 30 min. Steel beads showed a similar time scale, propagating as early as 2 h for 1 bead and within 1 h for 5 coated beads. Finally, Teflon^®^ beads induced the formation of detectable amounts of rec-PrP^res^ at 2 h irrespective of using 1 or 2 beads. These results allowed determination of a rec-PrP^res^ PMSA propagation time window for each type of bead where (1) propagation is not observed (as a control of the sensitivity of the process), (2) propagation is first detected (the time point at which propagation is detectable but not maximal) and 3) the shortest time in which propagation reached the maximum capacity or plateau. Thus, t1, t2, and t3, respectively, were established for each type of bead, dependent on their ability to propagate rec-PrP^res^ by PMSA and these time points were used to determine the success or otherwise of each decontamination treatment applied to the prion-coated beads of various material.

### Development of an Assay to Evaluate the Effect of Potential Prion Decontamination Procedures

**FIGURE 3:**
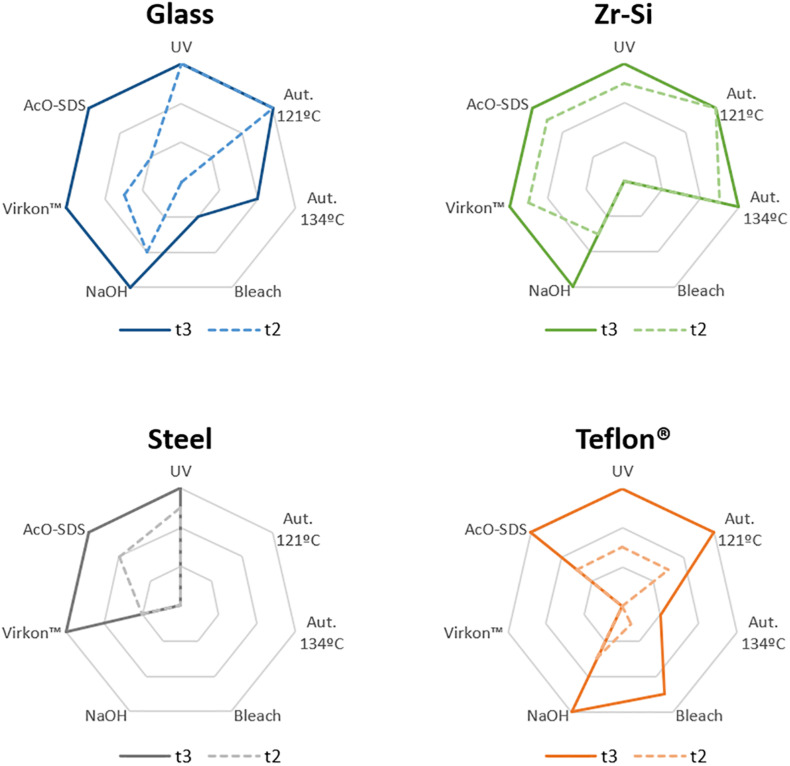
Graphical representation of the results obtained for rec-PrP^res^ propagation in PMSA from the different prion-coated beads treated with the chosen different decontamination procedures. Radar charts represent the number of replicate tubes subjected to PMSA showing rec-PrP^res^ by electrophoresis and total protein staining. Colored lines represent the presence of rec-PrP^res^, which indicates incomplete inactivation of the rec-PrP^res^ adsorbed to the surface of the beads. t2 (dotted lines) refers to the shortest PMSA reaction time in which rec-PrP^res^ was detected in the absence of any decontamination treatment but propagation has not reach a *plateau*, and t3 (solid lines) refers to the shortest PMSA reaction time in which rec-PrP^res^ signal reached *plateau* which is considered to be the maximum propagation limit. Each axes of the radar chart represent one of the decontamination treatment used that are detailed in Table 1 (autoclave at 121°C, at 134°C, NaOH, bleach, Virkon^TM^ and AcO-SDS treatment). The plots of the results of Table 2, show clearly that prions adsorbed to steel beads are the ones more easily eliminated by most of the treatments used, and also that bleach and autoclave at 134°C are the most effective for decontamination of prions from most of the surface materials tested, with the exception of Teflon^®^ beads highly susceptible to the treatment with Virkon^TM^.

**FIGURE 4:**
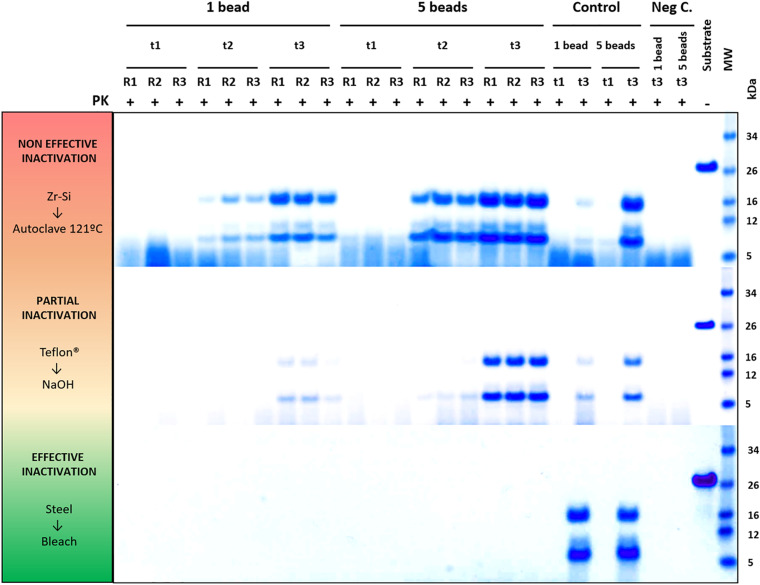
Representative decontamination treatment evaluation experiments showing examples of a non-efficient, partially efficient and completely efficient treatment for prion decontamination. Electrophoresis and total protein staining of the Proteinase K (PK)-digested PMSA products of three representative experiments are shown as examples of decontamination treatment evaluation by PMSA. Three replicates (R1, R2, and R3) of either 1 or 5 prion-coated zirconia-silica (Zr-Si) beads treated by autoclaving at 121°C for 20 min and submitted to a PMSA round at three time points (t1, t2, and t3), serve as an example of a non-effective inactivation treatment, since rec-PrP^res^ with the characteristic ∼16 kDa predominant band could be detected in all replicates at t2 and t3. Samples at t1 were negative which, being a time point chosen as a control of the process, indicates that the propagation kinetics were as expected. Positive control samples (Control), performed with untreated prion-coated beads of the same type at t1 and t3 demonstrate that the PMSA allowed propagation as expected, and negative controls (Neg C.), performed using non-coated beads of the same time show there was no cross contamination or spontaneous generation during the process. A sample of substrate, not digested with PK, was also included to facilitate size comparisons. As an example of a partially effective treatment, the experiment performed using Teflon^®^ beads treated with 1N NaOH for 1 h is shown. Finally, an example of a completely effective inactivation, the experiment carried out with prion-coated steel beads treated with commercial bleach for 1 h is shown. MW, Molecular weight marker.

To validate this assay, a panel of physicochemical decontamination procedures was selected and subjected to it. The different treatments were chosen based on WHO recommendations, previous reports or on treatments commonly used for prion decontamination in laboratories working on TSE (summarized in Table 1). To assess the effect of the treatments on the propagation capacity of prions, beads of each type were used after decontamination to seed PMSA reactions with different durations as determined in the previous section. The results corresponding to all beads and decontamination treatments are summarized in Table 2 and visually represented in Figure 3, although only different reaction times were considered given the identical results obtained with 1 or 5 beads (2 in the case of Teflon^®^) for the selected time points (raw data available at Supplementary Figure 2). Original electrophoresis gels of representative experiments are also shown as examples of the result readouts (Figure 4).

Complete decontamination (0 out of 6 replicates positive for rec-PrP^res^ at t3) of adhered rec-PrP^res^ was achieved after treatment of steel beads with bleach, NaOH or in the autoclave, either at 121 or 134°C for 20 min. It was also achieved after treating zirconia-silica beads with bleach and Teflon^®^ beads with Virkon^TM^. In all the other cases prion inactivation was incomplete but bleach was the most effective decontaminant for most type of beads followed by autoclaving at 134°C. Prions adsorbed to glass beads, with two positive tubes at t3, and Teflon^®^ beads, with 4 out of 6 positive tubes, resisted inactivation by bleach, most likely due to the physical properties of the bead material. As expected, UV treatment showed no effect on prion inactivation causing slight reduction of prion propagation at t2 for zirconia-silica, steel and Teflon^®^ beads. Similarly, acidic SDS treatment also showed little effect reducing prion propagation at t2, but not achieving complete inactivation in any case. Surprisingly, Virkon^TM^, a well-known virucidal agent showed some effect on prion propagation capacity at t2 for all the types of beads, providing total prion inactivation in the case of Teflon^®^ beads. Finally, the treatment with 1N NaOH showed lower prion inactivation capacity than expected for zirconia-silica, glass and Teflon^®^ beads, inhibiting propagation in few replicates just at t2 and suggesting that the effect of the decontaminant treatment is highly dependent on the treated surface material.

### Combination of Decontamination Treatments With Non-ionic Surfactants Can Improve Prion Inactivation Efficiency in Hydrophobic Surfaces

The unexpectedly low prion inactivation capacity showed by bleach and NaOH treatments for some of the surface materials used such as Teflon^®^, prompted us to investigate whether it was due to hydrophobicity or surface tension that could avoid proper contact between the decontaminants and the prions adsorbed to the surface of this material. For that, 1% Triton-X-100, a non-ionic detergent, was added to the original treatments with commercial bleach and 1N NaOH. The results summarized in Table 3 clearly demonstrate that the addition of Triton-X-100 greatly improved the inactivation of prions coating Teflon^®^ beads treated with bleach or NaOH, achieving complete decontamination.

## Discussion

Protein misfolding shaking amplification (PMSA) has been already shown to propagate recombinant bona fide prions in a simple but highly efficient and robust way (Erana et al., 2019). Requiring a minimum of laboratory equipment, a shaker with speed and temperature control, the scalability and the robustness of the system makes it highly versatile as it is able to generate large amounts of recombinant prions and perform high-throughput screenings of potential prion decontamination procedures or anti-prion drugs. Taking advantage of the ability of recombinant prions to adsorb on to the surface of beads of different materials and the exceptional sensitivity of PMSA to detect their presence, we have developed a method capable of assessing the prion inactivation capacity of distinct physicochemical treatments. Using decontaminants recommended by the WHO or CDC (WHO, 1999; CDC, 2019), along with other treatments known to have little effect or no effect on prion inactivation, we have validated this novel method for this purpose.

Although most published studies on prion decontaminants rely on bioassays to evaluate their efficacy, in terms of infectivity (Taylor, 2000), some *in vitro* (Fichet et al., 2004; Belondrade et al., 2016, 2020; Hughson et al., 2016; Mori et al., 2016; Gough et al., 2017) and *ex vivo* (Edgeworth et al., 2011; Botsios et al., 2015) methodologies have been used to assess the efficacy of treatments and methods based on highly sensitive *in vitro* prion propagation systems have been considered most successful. However, decontamination treatments applied on samples with low prion titers or using model systems with not enough sensitivity for prion detection can dangerously overestimate prion elimination capacity of a treatment (Giles et al., 2017). Despite the reported success of highly sensitive *in vitro* methods, without bioassay they cannot guarantee complete absence of infectious capacity and only provide an approximation based on assessment of propagation capacity for which only minute amounts of infectious prions are required. Therefore, although initial studies on different decontamination procedures were mostly performed through treatment of prion-containing brain homogenates and inoculation in model animals, the improvement of *ex vivo* and *in vitro* models is starting to gain importance. Apart from the obvious benefits in terms of cost, turnaround time and animal welfare of the *in vitro* techniques, the availability of permissive animal models for each prion strain under analysis is also a significant limitation of the bioassay. Giles and collaborators highlighted the importance of evaluating the efficacy of a decontamination treatment for a particular prion strain by showing that bovine spongiform encephalopathy (BSE) prions are 1,000-fold more resistant to inactivation than the mouse-adapted BSE strain 301V, and up to a million-fold more resistant than some other strains examined (Giles et al., 2008). However, *in vitro* methods for evaluation of decontamination efficacy allow using as many different strains as desired thereby increasing their versatility as shown by Hughson et al. using RT-QuIC to examine the efficacy of decontaminants against five different prion strains concurrently (Hughson et al., 2016). For our PMSA-based method we chose a recombinant prion derived from bank voles, the infectivity of which was determined previously along with its biochemical characteristics (Erana et al., 2019). Specifically, its high PK-resistance is noteworthy [resistant to more than 1,000 μg/ml of PK which is equivalent or even higher than BSE, 1,000 μg/ml of PK but as whole brain extract, while the recombinant prion contains only dextran (Masujin et al., 2008)], indicating a highly packed amyloid core that, theoretically, will confer exceptional resistance to decontamination treatments. Therefore, using experimentally obtained recombinant prions to provide consistency to this novel method, although not directly related to natural mammalian-derived strains, is offset by using recombinant prions showing the most resistance to destruction. Although only Sst01 prions were used in this study, we have already generated several distinct rec-PrP^res^ isoforms showing different characteristics, using recombinant PrP from several mammalian species. All these recombinant prion conformers could be used together in the assay, providing exceptional robustness and reliability and therefore safely representative of less stable tissue-derived prions.

A critical advantage of this novel assay is the use of dried beads of different materials as prions can be adsorbed to the surfaces of many different materials that have been washed and dried. This approach has been used previously, principally by coating steel wires with prions by incubation with brain homogenates from affected animals. These prion-coated steel wires have been used to assess the efficacy of prion inactivation or elimination treatments by determining infectivity *in vivo* (Peretz et al., 2006; Giles et al., 2008; Berberidou et al., 2013) and by other criteria by *ex vivo* (Edgeworth et al., 2011) and *in vitro* (Fichet et al., 2004; Belondrade et al., 2016; Mori et al., 2016; Belondrade et al., 2020) methodologies also. Limited studies have been performed using other materials, mainly metallic or oxidized surfaces such as titanium (Berberidou et al., 2013), aluminum oxide and silicon dioxide (Jacobson et al., 2013), and non-metallic materials such as bricks (Gough et al., 2017) or farm soil (Sohn et al., 2019). These data, together with the results presented here, clearly demonstrate the relevance of the material prions are attached to with respect to the variability in efficacy of decontaminants, highlighting differences even between metallic surfaces such as steel and titanium (Berberidou et al., 2013). However, the ability to use prion-coated materials for assessment of decontaminant procedures after washing and drying allows greater representation of the real situations where prions could be found. Dehydration may promote and increase in prion resistance to inactivation or elimination (Yuan et al., 2018), so the use of brain homogenates or prions recovered from contaminated surfaces in solvents for the evaluation of decontamination may significantly overestimate their efficacy. The novel method presented here is the first reported that examines several radically different surface materials mimicking the real-life scenario including exposure to being washed and dried previously to replicate what effect this may have on adhered prions. The major weaknesses of decontaminant assessment methodologies based exclusively on *in vitro* prion propagation is the lack of direct comparison with confirmatory *in vivo* assays as correlation between rec-PrP^res^ detection and prion infectivity would be ideal. The primary aim of this study was to report a low-cost, easy and fast assay that could be used as a pre-screening assay to evaluate many different decontamination treatments and bioassay would only be required for those treatments that are successful on *in vitro* prion propagation. However, in our extensive experience with *in vitro* prion propagation methods, we have never observed infectivity in bioassay from a recombinant seed that had been unable to give rise to rec-PrP^res^ after being submitted to PMSA, suggesting a good correlation between *in vitro* propagation capacity and *in vivo* infectivity. Although, the possibility of a sample retaining infectivity *in vivo* despite being undetectable by PMSA propagation cannot be excluded completely, this has been addressed previously by others who showed good correlation as long as the *in vitro* amplification technique was highly sensitive (Lee et al., 2000; Belondrade et al., 2020). Therefore, given the data, we consider our novel system will have excellent correlation with rec-PrP^res^ detection by bioassay.

Our main goal was to develop an assay to assess potential prion decontamination treatments, not the evaluation of decontaminants *per se*. However, the results obtained with the treatments chosen and used as proof of concept for this methodology are noteworthy. The use of an autoclave at 121°C and UV irradiation were selected because they have been proven to be inefficient for prion inactivation (Alper et al., 1967; WHO, 1999; Taylor, 2000) and our results support this. Exposure to UV light did not reduce prion propagation capacity at t3 for any of the materials tested and, except for prions adsorbed to steel beads, autoclaving at 121°C showed little, if any, effect. The fact that prion-coated steel beads could be inactivated completely by this procedure could be due to a more significant reduction in propagative units than for other materials, it is possible that the steel beads already contained lower initial amount of prions or that the temperature resulted more effective eliminating prions from this surface due to its higher thermal conductivity, since a reduction in infectivity at 121°C have also been reported for brain homogenates (Prusiner, 1984; Ernst and Race, 1993). In fact, autoclave at 121°C has been previously reported as partially efficient for the decontamination of prion-coated metal wires, evaluated by PMCA (Belondrade et al., 2016). Whether this increased reduction of prions adsorbed to steel beads in comparison with the rest is a result of a lower load of prions, unlikely according to preliminary propagation experiments, or a different mechanism of prion-surface interaction cannot be explained at present. On the contrary, the autoclave at 134°C, bleach and NaOH treatments were chosen due to their efficacy, according to the WHO, and, as such, they are used routinely in research laboratories working with prions (WHO, 1999). Surprisingly, the autoclave method was only partially effective for most of the materials examined, being especially ineffective for zirconia-silica and glass beads, which may have the most irregular or porous surfaces among the materials used, suggesting some degree of protection for prions adsorbed to such materials. This partial decontamination by autoclaving at 134°C has also been reported for other systems based on steel wires and bioassay, revealing the importance of humidity during the process (Fichet et al., 2004) and which could explain our results. NaOH and bleach are the most popular and widely used chemical decontaminants and bleach was the most effective decontaminant agent for prions by our assay. However, striking differences were found depending on the bead materials used. In the case of steel beads, decontamination was complete in agreement with previous results obtained *in vitro* and *in vivo* with brain homogenates and steel wires (Fichet et al., 2004; Lemmer et al., 2008; Hughson et al., 2016), although for other materials results can differ as demonstrate by the work of Gough et al. in which NaOH and free chlorine were unable to decontaminate prions on the surface of bricks (Gough et al., 2017). Therefore, we can assume that the characteristics of materials such as zirconia-silica, glass or Teflon^®^ can limit the effectiveness of bleach and NaOH treatments either by protecting prions inside the pores of a coarse surface or hindering proper contact between the decontaminant solution and the prions due, for example, to hydrophobicity. The results obtained for Teflon^®^ beads with Virkon^TM^, a well-known virucidal with abundant peroxides and surfactants with unknown effect on prions, and acidic SDS treatment, proved partially effective (Peretz et al., 2006), resulted in similar or even better results than bleach and NaOH. This prompted us to investigate whether it was due to hydrophobicity or surface tension that was hindering proper contact between the decontaminants and the prions adsorbed to the surface of this material. The presence of Triton-X-100 in the bleach or NaOH solutions greatly improved their prion elimination capacity, indicating the importance of the properties of each material with bound prions when assessing decontamination treatments. The presence of a non-ionic surfactant probably facilitates the access of the decontaminants to the highly hydrophobic surface of the bead, although other mechanisms cannot be ruled out.

Overall, we present a novel PMSA method to assess prion decontamination or inactivation procedures suitable for examining prions bound to different materials’ surfaces that is easy to perform in any laboratory using minimum equipment and biosafety conditions. By using known effective, partially effective or ineffective treatments, we have validated this method with previous results. Furthermore, as previous results were obtained mainly by treatment of brain homogenates or prions bound to steel wires, we have provided novel data and new insights with respect to the decontamination of surface adsorbed prions, revealing the relevance that different surface materials and their possibly distinct interactions with prions influence significantly the efficacy of different prion decontamination treatments. Combinations of different treatments that increase their efficacy are actually recommended (WHO, 1999) and when used have reduced the risk of iatrogenic transmission of prion disorders almost completely. However, in order to find a universal decontamination treatment effective for any kind of prion strain bound to any surface, assessment methods such as the novel one presented here are required given the continued threat to public health posed by extremely persistent prions.

## Data Availability Statement

All datasets generated for this study are included in the article/Supplementary Material, further inquiries can be directed to the corresponding author.

## Author Contributions

HE, MP-C, SG-M, and JC contributed to the conception and design of the study. MP-C, SG-M, JMC, EG-M, RL-M, CD-D, and TB performed the experiments, interpreted the results, and designed figures and tables. HE, MP-C, and TB wrote the first draft of the manuscript. JC was responsible for funding acquisition. All authors contributed to manuscript revision, read, and approved the submitted version.

## Conflict of Interest

HE, JMC, SG-M, and EG-M were employed by the commercial company ATLAS Molecular Pharma SL. This does not alter our adherence to all Frontiers policies on sharing data and materials. The remaining authors declare that the research was conducted in the absence of any commercial or financial relationships that could be construed as a potential conflict of interest.
