# Supplementary material for: A Novel, Reliable and Highly Versatile Method to Evaluate Different Prion Decontamination Procedures
Source: Front Bioeng Biotechnol. 2020 Oct 29;8:589182. doi: 10.3389/fbioe.2020.589182 (PMC7658626; doi:10.3389/fbioe.2020.589182)
Supplement: Supplementary file 1 [file Data_Sheet_1.DOCX]

**SUPPLEMENTARY MATERIAL**

**Supplementary figure 1. Electrophoresis and total protein staining of fractions collected during recombinant bank vole PrP expression and purification to monitor the process.** During bacterial expression and purification of recombinant bank vole prion protein used in all the experiments presented, fractions were collected to monitor the process. 10 µl of each fraction, Lysis, Loading, Washing and Elution were collected as indicated in Materials and Methods section, precipitated with cold methanol and submitted to electrophoresis and total protein staining. Lysis fraction shows a prominent ~ 23 kDa band that corresponds to over-expressed bank vole PrP, which was observed with increased purity in Elution fraction. MW: Molecular weight marker.

**
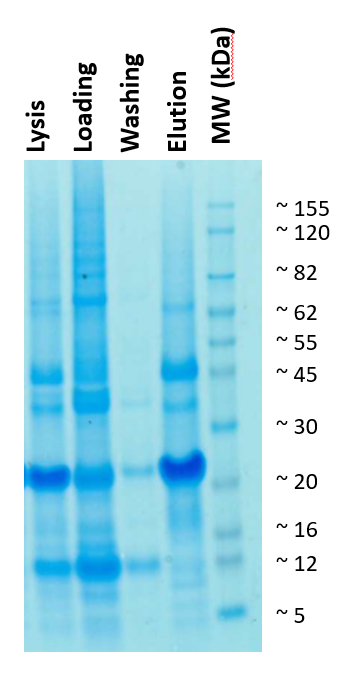
**

**Supplementary figure 2. Tables summarizing the raw data for the preliminary experiments and each decontamination treatment performed with the 4 bead types.** Number of tubes showing rec-PrP^res^ after PK digestion, electrophoresis and total protein staining is shown in each table out of the three replicate tubes used in each case. Preliminary data tables include the raw data graphically represented in figure 2, showing propagation of prion-coated beads of each type at different time points, using both 5 and 1 beads (2 and 1 beads in the case of Teflon®. Raw data of decontamination treatment experiments also contains the information of rec-PrP^res^ positive tubes at each time point. Zr-Si: Zriconia-sillica; AcO-SDS: Acidic-SDS treatment.

***Preliminary data***

|  | **Zr-Si** | |
| --- | --- | --- |
| **Time (h)** | **1 bead** | **5 beads** |
| **0.5** | 0 | 2 |
| **1** | 0 | 3 |
| **2** | 3 | 3 |
| **4** | 3 | 3 |
| **6** | 3 | 3 |
| **8** | 3 | 3 |

|  | **Glass** | |
| --- | --- | --- |
| **Time (h)** | **1 bead** | **5 beads** |
| **0.5** | 0 | 3 |
| **1** | 0 | 3 |
| **2** | 3 | 3 |
| **4** | 3 | 3 |
| **6** | 3 | 3 |
| **8** | 3 | 3 |

|  | **Teflon®** | |
| --- | --- | --- |
| **Time (h)** | **1 bead** | **2 beads** |
| **0.5** | 0 | 0 |
| **1** | 0 | 0 |
| **2** | 1 | 3 |
| **4** | 3 | 3 |
| **6** | 3 | 3 |
| **8** | 3 | 3 |

|  | **Steel** | |
| --- | --- | --- |
| **Time (h)** | **1 bead** | **5 beads** |
| **0.5** | 0 | 0 |
| **1** | 0 | 3 |
| **2** | 3 | 3 |
| **4** | 3 | 3 |
| **6** | 3 | 3 |
| **8** | 3 | 3 |

***Decontamination experiments***

|  | **Autoclave 121ºC** | | | | | |
| --- | --- | --- | --- | --- | --- | --- |
|  |  | **1 bead** |  |  | **5 beads** |  |
|  | **t1** | **t2** | **t3** | **t1** | **t2** | **t3** |
| **Glass** | 0 | 3 | 3 | 0 | 3 | 3 |
| **Zr-Si** | 0 | 3 | 3 | 0 | 3 | 3 |
| **Steel** | 0 | 0 | 0 | 0 | 0 | 0 |
| **Teflon®** | 0 | 0 | 3 | 0 | 3 | 3 |

|  | **Autoclave 134ºC** | | | | | |
| --- | --- | --- | --- | --- | --- | --- |
|  |  | **1 bead** |  |  | **5 beads** |  |
|  | **t1** | **t2** | **t3** | **t1** | **t2** | **t3** |
| **Glass** | 0 | 0 | 1 | 0 | 0 | 3 |
| **Zr-Si** | 0 | 2 | 3 | 0 | 3 | 3 |
| **Steel** | 0 | 0 | 0 | 0 | 0 | 0 |
| **Teflon®** | 0 | 0 | 0 | 0 | 0 | 2 |

|  | **Bleach** | | | | | |
| --- | --- | --- | --- | --- | --- | --- |
|  |  | **1 bead** |  |  | **5 beads** |  |
|  | **t1** | **t2** | **t3** | **t1** | **t2** | **t3** |
| **Glass** | 0 | 0 | 0 | 0 | 0 | 2 |
| **Zr-Si** | 0 | 0 | 0 | 0 | 0 | 0 |
| **Steel** | 0 | 0 | 0 | 0 | 0 | 0 |
| **Teflon®** | 0 | 0 | 2 | 0 | 1 | 3 |

|  | **NaOH** | | | | | |
| --- | --- | --- | --- | --- | --- | --- |
|  |  | **1 bead** |  |  | **5 beads** |  |
|  | **t1** | **t2** | **t3** | **t1** | **t2** | **t3** |
| **Glass** | 0 | 2 | 3 | 0 | 2 | 3 |
| **Zr-Si** | 0 | 0 | 3 | 0 | 3 | 3 |
| **Steel** | 0 | 0 | 1 | 0 | 0 | 0 |
| **Teflon®** | 0 | 0 | 3 | 0 | 2 | 3 |

|  | **Virkon™** | | | | | |
| --- | --- | --- | --- | --- | --- | --- |
|  |  | **1 bead** |  |  | **5 beads** |  |
|  | **t1** | **t2** | **t3** | **t1** | **t2** | **t3** |
| **Glass** | 0 | 2 | 3 | 0 | 1 | 3 |
| **Zr-Si** | 0 | 2 | 3 | 0 | 3 | 3 |
| **Steel** | 0 | 1 | 3 | 0 | 1 | 3 |
| **Teflon®** | 0 | 0 | 0 | 0 | 0 | 0 |

|  | **UV** | | | | | |
| --- | --- | --- | --- | --- | --- | --- |
|  |  | **1 bead** |  |  | **5 beads** |  |
|  | **t1** | **t2** | **t3** | **t1** | **t2** | **t3** |
| **Glass** | 0 | 3 | 3 | 0 | 3 | 3 |
| **Zr-Si** | 0 | 1 | 3 | 0 | 3 | 3 |
| **Steel** | 0 | 2 | 3 | 0 | 3 | 3 |
| **Teflon®** | 0 | 0 | 3 | 0 | 3 | 3 |

|  | **AcO-SDS** | | | | | |
| --- | --- | --- | --- | --- | --- | --- |
|  |  | **1 bead** |  |  | **5 beads** |  |
|  | **t1** | **t2** | **t3** | **t1** | **t2** | **t3** |
| **Glass** | 0 | 1 | 3 | 0 | 0 | 3 |
| **Zr-Si** | 0 | 2 | 3 | 0 | 3 | 3 |
| **Steel** | 0 | 1 | 3 | 0 | 3 | 3 |
| **Teflon®** | 0 | 1 | 3 | 0 | 2 | 3 |

|  | **Bleach + Triton X-100** | | | | | |
| --- | --- | --- | --- | --- | --- | --- |
|  |  | **1 bead** |  |  | **5 beads** |  |
|  | **t1** | **t2** | **t3** | **t1** | **t2** | **t3** |
| **Teflon®** | 0 | 0 | 0 | 0 | 0 | 0 |

|  | **NaOH + Triton X-100** | | | | | |
| --- | --- | --- | --- | --- | --- | --- |
|  |  | **1 bead** |  |  | **5 beads** |  |
|  | **t1** | **t2** | **t3** | **t1** | **t2** | **t3** |
| **Teflon®** | 0 | 0 | 0 | 0 | 0 | 0 |
